# Development and Implementation of an Evidence-Based Electronic Request Bundle for Metabolic Screening in Patients With Urinary Tract Calculi

**DOI:** 10.7759/cureus.73448

**Published:** 2024-11-11

**Authors:** Zakaria Saidani, Shaswath Ganapathi, Salim S Malik, Fahd Khan

**Affiliations:** 1 Urology, Sandwell and West Birmingham Hospitals, Birmingham, GBR

**Keywords:** electronic requesting, health technology, metabolic screening, nephrolithiasis, urology

## Abstract

Introduction

Kidney stone disease is a global health issue, with a well-known high recurrence rate after a first stone episode. Metabolic screening is a cost-effective tool for identifying patients at risk of recurrence due to a secondary disease. Our study aimed to assess compliance in a District General Hospital (DGH) and lay out a framework for the implementation of an electronic request bundle to improve screening levels in line with National Institute for Health and Care Excellence (NICE) guidelines.

Methods

A retrospective, cross-sectional study was conducted on 67 eligible patients, presenting with nephrolithiasis between May 2023 and July 2023. The percentage undergoing metabolic screening on admission was recorded and compared against previous Trust data. Clinical staff were surveyed to develop a streamlined electronic bundle, which was then implemented across the Trust collaboratively with Unity EPR engineers and senior clinical and non-clinical management.

Results

Thirty (47.8%) eligible patients had serum calcium levels measured on admission, and 25 (37.3%) patients had serum urate measured - similar screening levels to that collected during previous audits in the same Trust. Of the 30 surveyed staff, 12 (40.0%) reported they would routinely check serum urate and 15 (50.0%) serum calcium in renal stone presentations. In total, eight months of stakeholder meetings were required to effectively develop and deploy a new electronic requesting bundle, aiming to increase metabolic screening levels at the Trust.

Discussion

Adherence to NICE guidelines in renal stone patients at this DGH was significantly below the recommended standards of care. Identifying intervention points, surveying staff, and working with key stakeholders allowed for the integration of a metabolic screening tool. This study provides an effective framework for the development and sustainable implementation of an electronic requesting bundle, which can be modeled healthcare-wide.

## Introduction

Renal colic is one of the most common elective and emergency urological presentations, affecting up to 20% of men and 10% of women in their lifetime [1-3]. Not only is the economic burden of renal stone disease vast, but the direct cost to the NHS is substantial, with an estimated cost in 2010 of between £190 million and £324 million. This is comparable to the combined cost of prostate and bladder cancer in the UK [3]. For most individuals, nephrolithiasis etiology is a combination of both genetic and environmental factors, with twin studies suggesting heritability of >45% [4]. Recent evidence suggests that renal stone disease linked to dietary and metabolic risk factors is on the increase, especially in relation to uric acid stones [5,6]. Given the likely increasing culpability of environmental factors in the development of renal stones, as well as the chronicity of nephrolithiasis, it is important to highlight the important value that metabolic screening can play in reducing the risk of secondary presentations in renal stone disease.

Though little data exists within the NHS regarding UK-wide adherence to metabolic screening guidelines, Milose et al. (2014) demonstrated that only 7.4% of patients in the United States with a kidney stone episode underwent metabolic evaluation in line with American Urological Association guidance [7]. National Institute for Health and Care Excellence (NICE) guidelines stipulate that serum calcium should be measured for adults with ureteric or renal stones [5] whilst the European Association of Urology (EUA) also recommends uric acid should be screened [8].

This study evaluated the levels of metabolic screening of renal stone disease patients and how this can be improved with the use of an electronic requesting bundle. Additionally, hurdles associated with the implementation of new technologies within healthcare were evaluated, such as an electronic requesting bundle, and the methods for overcoming this were concluded.

Over the past decade, with the widespread adoption of electronic health records, there has been increased research into the effectiveness of electronic detection systems and care bundles [9]. However, enacting change within healthcare, especially integrating technological innovation, can be a very convoluted, difficult process [10-12]. Despite digital transformation aiming to improve patient experience and outcomes, enacting change within healthcare can be vastly hurdled [11].

This study works to outline the methodology and interventions used to overcome obstacles in enacting change within healthcare, with the example of the real-life development of an electronic requesting bundle. Many changes within healthcare fail to achieve their desired goals, with a failure rate of around 70% cited [12]. In this study, Kotter’s eight stages of change model were used to plan, develop, and successfully implement a metabolic screening electronic bundle, building on existing literature on the complex process of the novel implementation of health technology.

The results of this study will provide a template that can act as a guide not only across urology departments UK-wide for developing and implementing electronic requesting bundles but also condition-wide.

This article was previously presented as a poster at the ASiT 48th Annual Conference, 9th - 10th March 2024; and the West Midlands Surgical Society Conference 2024, 24th of May 2024.

## Materials and methods

Metabolic screening re-audit

A retrospective, observational study was conducted on patients presenting with nephrolithiasis between May 2023 and July 2023. First, an extended data search was conducted via patient lists obtained from the urology department secretaries of unfiltered patients attending outpatient follow-up nephrolithiasis clinics, held weekly, between July and November 2023. A manual search of the lists was undertaken, and data collection was split between three clinicians. Data collection was approved by the Clinical Effectiveness Team and registered on the Trust Audit Database (ID 2439).

Inclusion and exclusion criteria are summarized in Table 1.

**Table 1:** Inclusion and exclusion criteria set out prior to data collection The single hyphen represents an empty cell.

Inclusion criteria	Exclusion criteria
Patients who had presented only between May and July 2023 within the data set.	Those previously diagnosed with treated hypercalcemia or hyperuricemia at any point.
Must have presented through the local accident and emergency department.	Patients who had presented with nephrolithiasis either once within the past six months, or more than twice in the preceding year.
Confirmed renal tract stone on CT imaging.	Those under the age of 18.
-	Referrals from Primary Care.

Data Collection

Pre-specified data were collected, including age; whether serum calcium and urate had been measured; stone location; and when the stone clinic follow-up was. Data were recorded via a secure spreadsheet and compared against previous metabolic screening audits carried out at the Trust from 2021 to 2023.

Statistical Analysis

Data input and post-hoc analysis were undertaken using GraphPad Prism software (GraphPad Software, Boston, MA, US), including generated figures. Descriptive statistics were used to summarize the demographic and clinical characteristics of the eligible patients. Categorical variables, such as gender and age cohorts, were expressed as frequencies and percentages. Survey responses were analyzed using frequency distributions and percentages calculated from responses.

Pathway mapping

**Figure 1:**
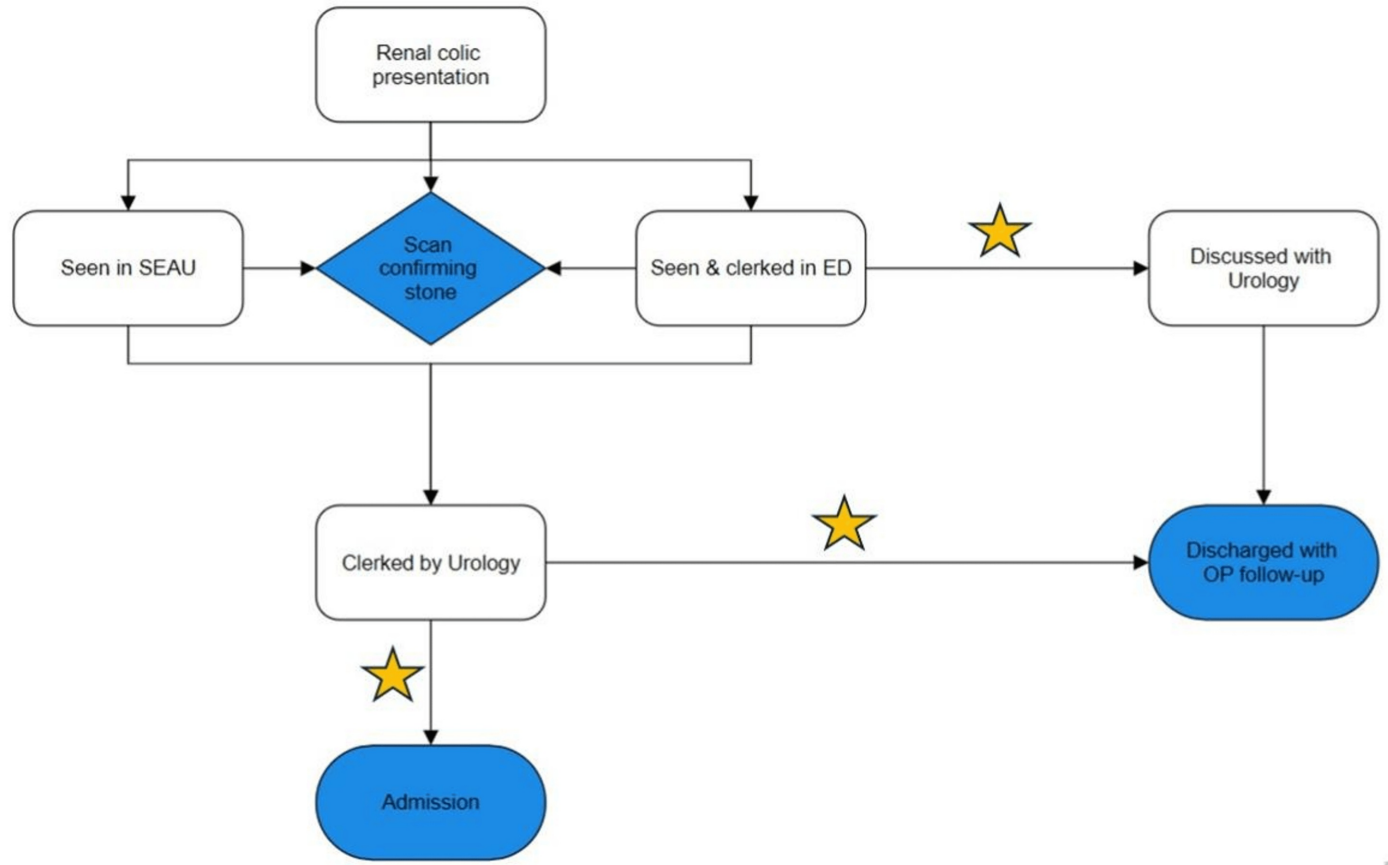
Flowchart summarizing potential pathways of a nephrolithiasis patient Gold stars indicate pathway opportunities for metabolic screening to be undertaken. Surgical Emergency Assessment Unit (SEAU), Emergency Department (ED), Outpatient (OP)

A process flowchart was developed, amalgamating the potential nephrolithiasis patient pathways, and the clinical staff they would come into contact with (Figure 1). Intervention opportunities at which metabolic screening would be best undertaken were identified, illustrated by the gold stars. A scoping review was then carried out using the Arksey and O'Malley framework to determine facilitators and barriers to the successful development, implementation, and evaluation of care bundles in the acute setting in the hospital. Key stakeholder clinical staff were then identified using this methodology and targeted with educational and questionnaire resources around the requesting bundle.

Staff survey

The Delphi method approach was used for the development of a questionnaire consisting of predominantly closed response items. This was circulated to front-line clinical staff involved in the care of a nephrolithiasis patient. Emails were circulated twice via clinical leads, and the survey window closed after one month. Dichotomous, semantic differential scales, and Likert scales were used to develop a five-question survey. Data were collected and analyzed via an online questionnaire generator. This data was used to develop the key elements of the electronic request bundle. The full questionnaire is listed in the appendix.

## Results

Patient demographics

A total of 67 eligible patients were identified, with the majority (45; 67.2%) being male, and females accounting for 22 (32.8%) patients. Patients were split into three age cohorts - 0-29 years old, 30-59 years old, and 60 years old plus. Forty-three (43; 64.2%) of patients were between the ages of 30-59, with the remaining split between 60+ years old, making up 15 (22.4%) of the patients, and 0-29 years old making up the remaining 9 (13.4%). Of the total patient population, it was the first recorded nephrolithiasis presentation for 44 (65.7%).

Metabolic screening

Of the 67 eligible patients, 32 (47.8%) had serum calcium measured on admission, 25 (37.3%) had serum urate, and 23 (34.3%) had both their serum calcium and urate recorded on the same admission. One (1.5%) patient had only their serum urate checked.

Data were compared against previous metabolic screening data collections completed from May 2021 to November 2022 (Table 2).

**Table 2:** Metabolic screening at the Trust from May 2021 to June 2023 This study's re-audit data is included under June 2023. Total patients: 72 (May 2021), 48 (May 2022), 26 (November 2022), 67 (June 2023)

Audit date	Serum calcium screening rate	Serum urate screening rate
May 2021	31 (43.1%)	35 (48.6%)
May 2022	25 (52.1%)	17 (35.4%)
November 2022	17 (65.4%)	12 (46.2%)
June 2023	32 (47.8%)	25 (37.3%)

Process framework

**Figure 2:**
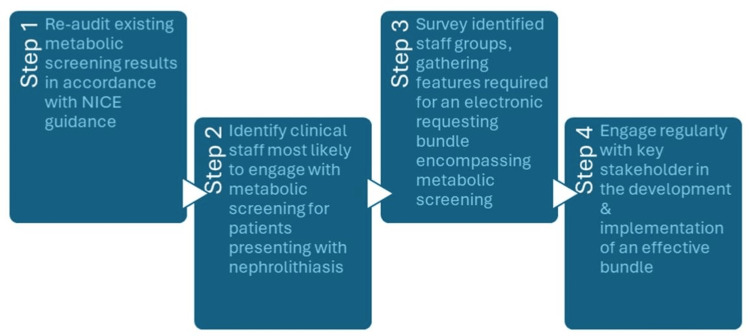
Summary of the framework process developed at the outset of the project under the guidance of senior urology department clinical staff

Utilizing Kotter’s eight stages of change model, a framework was mapped in order to have a targeted approach to developing and implementing an easy-to-use requesting bundle (Figure 2).

Survey results

A five-question survey was circulated to current front-line clinical staff involved in the early examination and workup of potential nephrolithiasis patients, encompassing both urology and emergency department staff of all levels. Thirty staff members completed the questionnaire.

**Figure 3:**
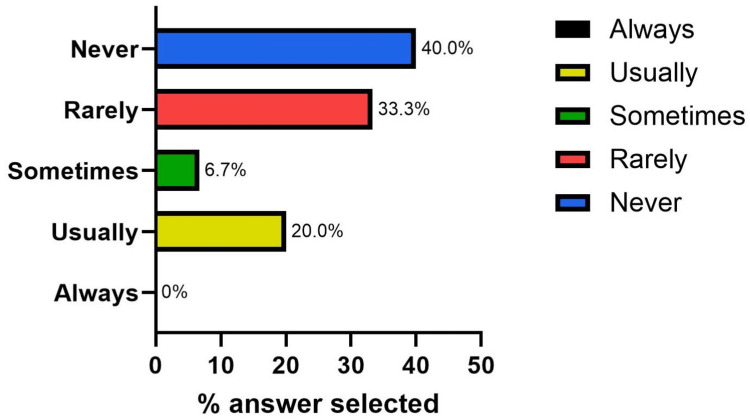
Answer breakdown: “How often do you use electronic request bundles?”

Previous electronic bundle use was first gathered with a single-answer question. No staff members responded that they "always" used electronic bundles for initial investigations, and 12 (40.0%) stated they "never" used a bundle (Figure 3).

**Figure 4:**
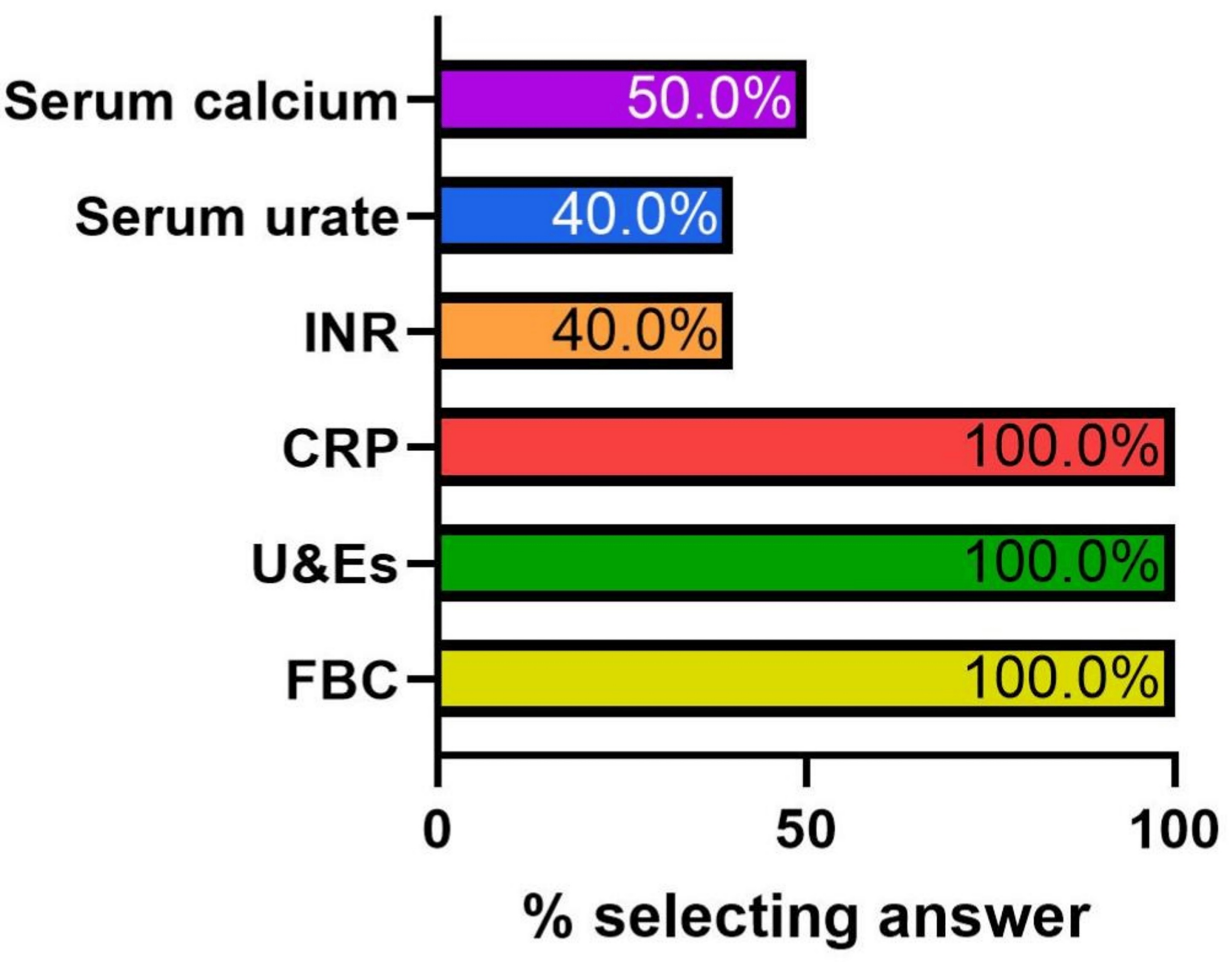
Answer breakdown: “Which blood tests would you order for a nephrolithiasis patient?” International normalized ratio (INR), C-reactive protein (CRP), urea and electrolytes (U&Es), full blood count (FBC)

Clinical staff were then asked a multiple-option answer question (Figure 4) regarding which blood tests they would order in patients presenting with nephrolithiasis. Fifteen (50.0%) responded they would order serum calcium and 12 (40.0%) serum urate. All participants answered they would order a full blood count (FBC), C-reactive protein (CRP), and urea and electrolytes (U&Es).

When asked if staff would be more likely to use a requesting bundle if it were easier to find and navigate, 77% answered yes.

**Figure 5:**
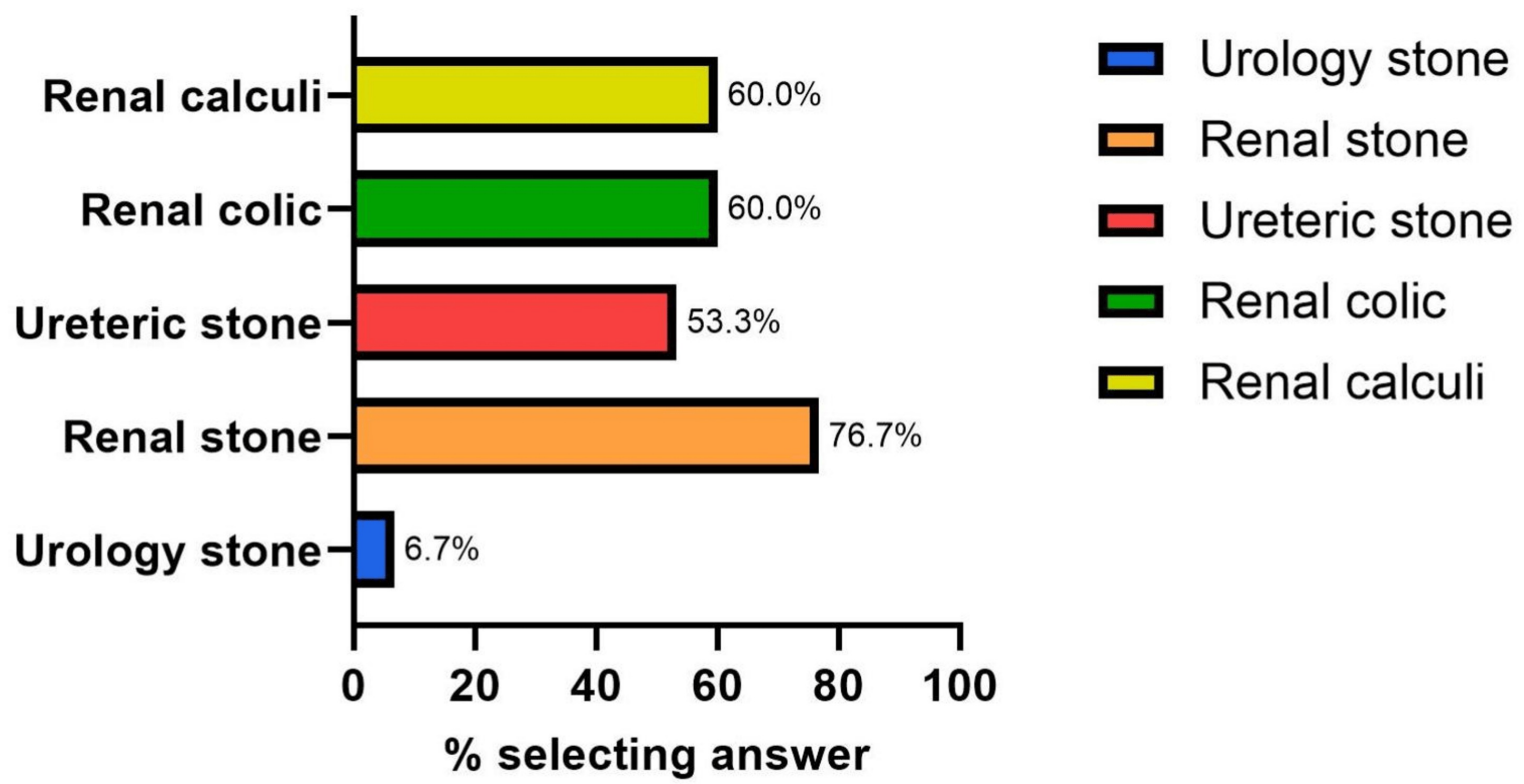
Answer breakdown: “Bundle title you would be most likely to search”

A multi-option question was utilized to ask clinical staff the term(s) they would be most likely to enter if searching for an initial investigation bundle for patients presenting with nephrolithiasis, with each respondent limited to only three answers maximum. The top three results were “renal stone”, with 23 (76.7%) responses; “renal colic”, with 18 (60.0%) responses; and “renal calculi” also with 18 (60.0%) cumulative responses (Figure 5).

## Discussion

Patients were categorized into three different age groups - 0-29 years old, 30-59 years old, and 60 years old plus. The median age category in our data was decided to be 30-59 years old to be in keeping with current evidence of the most commonly affected group; with the median age in the literature of those presenting with nephrolithiasis being 44.8 years old [11]. Our data also showed males were twice as likely compared to females to present with nephrolithiasis, in keeping with current literature conclusions showing a 2.43:1 male-to-female lifetime risk ratio [11]. The majority of patients included in our study were of a first-time nephrolithiasis presentation, 44 (65.6%) in total, which allowed for a more accurate reflection of metabolic screening on presentation rather than being influenced following outpatient specialist input. This was likely secondary to including exclusion criteria around repeat, short-timeframe re-presentations.

Re-audit data were then compared to the previous audit data from years prior. Analysis showed a consistent trend. Though simple interventions, such as educational posters, made limited improvements in metabolic screening, this improvement was not sustainable, and metabolic screening soon returned to baseline levels (Table 2). With the aim of enacting sustainable change to metabolic screening in nephrolithiasis patients, an electronic requesting bundle began to be developed. Literature on the topic suggests that the use of care bundles, coupled with the necessary education of key stakeholders directly involved in patient care, is the most effective method for enacting long-lasting change [9-10,13].

First, front-line clinical staff that were best placed to undertake metabolic screening needed to be identified. Mapping the potential streams a nephrolithiasis patient could enter helped identify areas where metabolic screening would be best placed to be carried out, as well as the key stakeholders in implementing this. Key stakeholders identified included: Emergency Department (ED) clinicians, ED triage nurses and phlebotomists, Surgical Emergency Assessment Unit (SEAU) clinicians, and SEAU nurses.

Following on from this, research suggested several features can hinder bundle integration. There is a negative association between the number of elements in a bundle and compliance [13]. The most successful implementation strategies involved the integration of key stakeholders and relationship building, coupled with education and training strategies [13].

It was deemed the most optimal preliminary step was to deploy a staff questionnaire to allow a bundle to be developed, which was inspired by the end users themselves, leading to an increased likelihood of its use. Literature searches on the topic concluded that, especially in conducting survey research among healthcare professionals, low response rates were directly correlated with increasing the number of survey questions [14]. This was likely secondary to the survey burden in healthcare, as well as time constraints faced by front-door clinical staff, reducing their likelihood of completing longer surveys [14]. Due to this, the number of questions was limited to a total of five, with a targeted approach aiming to improve response rates and data quality, particularly among busy professionals in clinical settings.

With the above potential barriers in mind, an initial questionnaire was extended to key stakeholders previously identified - the specific questions are summarized in the Appendices. Even assuming a well-justified and well-planned change initiative, research underscores the importance of managers building internal support for change by means of employee participation in the change process [10].

Results from the questionnaire suggested that the below-target level of metabolic screening was due to at least two main factors - the lack of knowledge that serum calcium and urate should be undertaken in nephrolithiasis patients and the general lack of use of requesting bundles. The questionnaire also helped identify key features that the bundle required; it needed to be easy to search for and needed to encompass the search terms “renal stone,” “renal colic,” and “renal calculi.”

Once developed, Kotter’s eight stages of change model was identified as a suitable framework for the implementation of the bundle and to ensure its use to become intrinsically associated with patients presenting with nephrolithiasis [15]. Although metabolic screening was clearly important to the urology department, a narrative needed to be put together to bring end users on board, as highlighted by van Gemert-Pijnen et al. [15]. Stakeholders from different backgrounds, with different interests and strategic influence, help create a cohesive team working unidirectionally [16].

On the requesting bundle becoming live, initial contact was made with the Clinical Effectiveness Team. Several emails and meetings were undertaken, and Microsoft PowerPoint (Microsoft Corporation, Redmond, WA, US) presentations delivered by the urology team highlighted key messages: 1) Metabolic screening was falling well below NICE guidelines, 2) The Trust's transition to becoming the Stone Centre for the region meant this was of pressing importance to address. Importantly, this helped align our clinical aims with managerial aims. The Clinical Effectiveness Team went on to arrange initial meetings with Clinical Service Programme Managers, Project Managers, and Implementation Teams. It is important to prioritize the values of different stakeholders and make choices based on this [16]. The end-zone of this led to collaborative joint meetings between our team (urology department), non-clinical managers, and medical and nursing clinical leads for both SEAU and the emergency department. The final agreed bundle components are illustrated (Table 3), with "bone profile" including serum calcium measured as standard.

**Table 3:** Final implemented components of the electronic request bundle for metabolic screening on Cerner Full blood count (FBC), urea and electrolytes (U&Es), C-reactive protein (CRP), prothrombin time (PT), computerized tomography kidneys, ureters, bladder (CT KUB) Single hyphens indicate empty cells. Cerner Corporation, North Kansas City, MO, US

Pre-selected	Not pre-selected
FBC	CT KUB
U&Es, blood	-
Urate level, blood	-
CRP, blood	-
PT, blood	-
Bone profile	-

Ultimately, a total of eight months of once-monthly stakeholder meetings, with interspersed email reminders and updates, allowed for the successful implementation of the electronic requesting bundle. Education in the form of physical posters and presentations at clinical staff inductions was provided around metabolic screening and the electronic request bundle, with additional relevant staff-wide email reminders sent by Clinical Leads. Most importantly, the relevant governance to ensure sustainable implementation has been put into place, with official changes to Trust-wide renal colic pathways now including metabolic screening using the electronic requesting bundle.

Bringing on-side non-clinical managers involved in service program implementation at the outset allowed for a unified approach when meeting with Clinical Leads. This was vital in ensuring that the agendas of all key stakeholders were aligned and that end users of the bundle received direct communication and education from their Leads.

The main contribution of this study lies in laying out the identified hurdles in implementing an electronic care bundle, with an evidence-based framework for overcoming these obstacles. While there is theoretical and literature-based research into the barriers and facilitators in successfully deploying health technologies, very little exists with the example of implementing a real-life electronic intervention within a healthcare setting. This can be adapted for broader clinical applications across different departments and conditions.

Study limitations

Small Sample Size

The study was conducted on 67 patients, limiting the generalisability of the findings, as well as the ability to carry out meaningful statistical analysis. However, this sample size did match that of the previous audits the data was compared to, so it allowed for adequate trends to be concluded. A larger sample size may have provided a more comprehensive understanding of metabolic screening rates.

Single-Center Study

Limiting data to only one Trust reduced the external validity of the findings, with practices, resources, and electronic patient record (EPR) systems varying throughout the NHS.

Survey Response Bias

The survey was conducted via an online questionnaire platform and communicated using email, with little in-person contribution. This may bias the responses slightly, with those more inclined to support and be comfortable with digital tools over-represented.

Barriers Not Fully Explored

While using the questionnaire did allow the study to identify and address some barriers to implementing and undertaking metabolic screening, such as ease of use and education, it did not comprehensively explore other potential barriers such as resource limitation, time constraints, or invariable resistance to change.

## Conclusions

In conclusion, this study highlights the need for improved adherence to metabolic screening guidelines in patients with nephrolithiasis and presents a successful approach to achieving this through the implementation of an electronic requesting bundle. The study's findings demonstrated that baseline metabolic screening rates for serum calcium and urate were significantly below NICE guidelines, underscoring the need for system-level changes to enhance compliance. By employing Kotter’s eight stages of change model and involving key stakeholders from both clinical and non-clinical backgrounds, the study successfully developed and integrated an electronic bundle into the hospital’s electronic patient records (EPR) system. This collaborative approach ensured that the bundle was user-friendly, easily searchable, and aligned with the needs of end-users. The eight months of structured stakeholder engagement and educational interventions were essential in overcoming barriers to adoption, ultimately resulting in a sustainable solution for improving patient care. This framework can serve as a guide for similar initiatives across other healthcare settings, demonstrating that with careful planning, collaboration, and education, long-term improvements in clinical practice can be achieved through the integration of digital tools in healthcare.

## Appendices

**Table 4:** All five questions included in the staff questionnaire Unity is the Trust Electronic Patient Record (EPR) system used

Questions included in the staff questionnaire:
1 - What bloods would you undertake in a patient presenting with a suspected first presentation of renal calculi? Select as many options as you agree with.
2 - How many of our patients do you estimate currently have their serum calcium and urate checked on first presentation with renal calculi?
3 - When searching for an initial investigation bundle on Unity for a first presentation of renal calculi, please select 3 phrases you would be most likely to search.
4 - How often do you use Unity bundles when requesting initial investigations in specific patient presentations?
5 - Do you believe you would be more likely to measure serum urate and calcium in renal calculi presentations if Unity bundles were easier to navigate/find?
